# Enhanced Cells Anchoring to Electrospun Hybrid Scaffolds With PHBV and HA Particles for Bone Tissue Regeneration

**DOI:** 10.3389/fbioe.2021.632029

**Published:** 2021-02-17

**Authors:** Joanna E. Karbowniczek, Łukasz Kaniuk, Krzysztof Berniak, Adam Gruszczyński, Urszula Stachewicz

**Affiliations:** Faculty of Metals Engineering and Industrial Computer Science, AGH University of Science and Technology, Kraków, Poland

**Keywords:** electrospinning, fibers, PHBV, hydroxyapatite (HA), tissue scaffold, bone regeneration, confocal microscopy, FIB-SEM

## Abstract

Hybrid materials combining organic and inorganic compounds used as scaffolds are highly beneficial in bone regeneration. In this study, we successfully produced by blend electrospinning poly(3-hydroxybutyric acid-co-3-hydrovaleric acid) (PHBV) scaffolds enriched with hydroxyapatite (HA) particles to biomimic bone tissue for improved and faster regeneration processes. The morphology, fiber diameters, and composition of the scaffolds were investigated by scanning electron microscopy (SEM) techniques followed by focused ion beam (FIB) sectioning to verify HA particles integration with PHBV fibers. *In vitro* cell culture was performed for 7 days and followed with the cell proliferation test (CellTiter-Blue^®^ Assay). Additionally, cell integration with the scaffold was visualized by confocal and SEM imaging. We developed a simple way of obtaining hybrid scaffolds by electrospinning PHBV solution with HA particles without any post-processing. The PHBV + HA scaffold enhanced cell proliferation and filopodia formation responsible for cell anchoring within the created 3D environment. The obtained results show the great potential in the development of hybrid scaffolds stimulating bone tissue regeneration.

## Introduction

The tissue regeneration process is strongly dependent on cell interaction with the materials used as a scaffold to enhance their growth and development. A widely exploited approach of tissue engineering aims to create functional structures in laboratory conditions that after implantation will replace, restore, or improve the functions of damaged or diseased organs. For this purpose, it combines cells, scaffolds, and signals in the form of growth factors or structural, mechanical, and electrical stimuli (Cassidy and Cartmell, 2013; Salinas et al., 2018). Designing the proper scaffold for each tissue type is crucial as it creates the whole microenvironment where cellular development takes place (Stevens and George, 2005). Bone tissue is a complex structure with a hierarchical arrangement of extracellular matrix (ECM) built of collagen type I and hydroxyapatite (HA) crystals. The presence of inorganic compounds ensures high strength, hardness, and rigidity essential for bones, whereas organic compounds provide flexibility and the reduction of brittleness (Alford et al., 2015). Altogether the structure and composition of ECM support the dynamic processes of bone formation and resorption during growth, remodeling, and healing (Filippi et al., 2020). To mimic the bone structure often hybrids or composite scaffolds, combining organic and inorganic components are prepared. This biomimetic approach in producing hybrid scaffolds has gained much attention over the last few years (Xing et al., 2019; Lyons et al., 2020). Therefore, various types of natural and synthetic polymers are studied in combination with bioactive ceramics to create osteoconductive scaffolds (Filippi et al., 2020; Lyons et al., 2020).

Highly porous composite scaffolds were prepared by different methods, e.g., solvent casting-particulate leaching (Baek et al., 2012; Wu et al., 2017), 3D printing (Huang et al., 2018), sponge replication followed by dip coating (Thomas and Bera, 2020), phase inversion technique (Podporska-Carroll et al., 2014), electrospinning (Suslu et al., 2014), and a combination of electrospinning and electrospraying (Ramier et al., 2014). Among these listed techniques electrospinning is especially interesting as it allows for the production in single-step hybrid meshes from a mixed polymer solution and ceramic particles. Therefore, in a simple, fast, and cost-effective way, it is possible to obtain suitable scaffolds for cardiac tissue engineering (Mani et al., 2019), neural guidance (Farzamfar et al., 2019), diabetic wound healing (Augustine et al., 2020), and bone regeneration (Bai et al., 2015; Sadat-Shojai, 2016; Kouhi et al., 2018).

The goal of producing hybrid scaffolds mimicking the ECM of bone is to obtain a supportive structure with tailored mechanical and surface properties. By using exposed bioactive compounds in the scaffolds we are able to promote cell anchoring and adhesion for further tissue development. Another aspect is related to material selection such as a piezoelectric polymer that can better biomimic bone tissue properties. The most studied piezoelectric polymers are poly(vinylidene fluoride) (PVDF) (Szewczyk et al., 2019a,b), poly(L-lactide) (PLLA), and poly(3-hydroxybutyric acid-co-3-hydrovaleric acid) (PHBV) (Lyons et al., 2020). Adding HA particles to bone tissue scaffolds (Fernandez-Yague et al., 2015; Yi et al., 2016) is often combined with piezoelectric and biodegradable polymers (Sultana and Wang, 2008). Apart from the most wildly studied bone scaffold material, polycaprolactone (PCL), another highly biocompatible material with desirable degradation time frames is PHBV (Chen and Wu, 2005). Previously, hybrid scaffolds based on PHBV electrospun fibers were immersed in simulated body fluids (SBF) for HA deposition (Ito et al., 2005), or directly prepared by electrospinning the suspension containing both the polymer and ceramic particles (Tong et al., 2010; Paşcu et al., 2013; Kouhi et al., 2015; Brunetti et al., 2020). Various properties of such scaffolds were studied, including: degradation (Ito et al., 2005), bioactivity and mechanical properties (Paşcu et al., 2013; Kouhi et al., 2015) as well as *in vitro* biocompatibility (Tong et al., 2010). These studies showed a high potential of hybrid scaffolds based on electrospun PHBV fibers with HA particles for tissue engineering.

Our unique studies take a single-step approach by electrospinning PHBV mixed with HA particles including the direct characterization of particle distribution within fibers and scaffolds by using advanced microscopy techniques. Therefore, in this study, we aimed to prove that hybrid scaffolds based on electrospun PHBV fibers enriched with HA particles enhance bone regeneration processes better in comparison to solely PHBV scaffolds. Morphology, fiber diameters, and composition of the scaffolds were investigated by scanning electron microscopy (SEM) techniques followed by focused ion beam (FIB) sectioning to expose fiber interiors, providing an excellent approach to show the incorporation of HA particles inside the PHBV fibers and their presence on the fibers’ surface. *In vitro* osteoblasts culture was incubated for 7 days to verify their proliferation with a CellTiter-Blue^®^ Assay. Additionally, cell integration with the scaffold was visualized by confocal and SEM imaging. We confirmed the beneficial effect of hybrid scaffolds on osteoblasts adhesion, spreading, proliferation, and filopodia formation through a combination of fibers’ topography and composition.

## Materials and Methods

### Materials and Scaffolds Preparation

Poly(3-hydroxybutyric acid-co-3-hydrovaleric acid) (PHV content 2 wt%, M_*w*_ = 450,000 g⋅mol^–1^, Helian Polymers, Netherlands) was dried before solution preparation for 4 h at 40°C. Two types of solutions were prepared: 8% PHBV and a blend of 8% PHBV with 1% of HA (particles size <200 nm, Sigma-Aldrich, United Kingdom). For the PHBV solution, 8 wt% of the polymer was dissolved in a chloroform and dimethylformamide (DMF) mixture (9:1, v/v, both solvents Sigma-Aldrich, United Kingdom). For the solution PHBV+HA, 0.1 g of HA particles were dispersed in the chloroform:DMF (9:1 v/v) mixture in an ultrasonic bath (Bandelin, Germany) for 10 min, and subsequently, 0.8 g of PHBV was added. Both solutions were stirred on a heated magnetic stirrer (IKA, Germany) for 4 h at 45°C until complete dissolution of the polymer. The solution containing nanoparticles was again ultrasonicated for 10 min prior to electrospinning. Both solutions were electrospun using an EC-DIG device with a climate control system (IME Technologies, Netherlands) at 25°C and RH = 40%. A voltage of 17 kV was applied to the stainless needle with an inner diameter of 0.8 mm, keeping a 20 cm distance to the grounded collector, the flow rate was 0.1 ml⋅min^–1^ for both solutions. Two types of scaffolds were produced: solely polymer - named PHBV, and the hybrid, which combines polymer and ceramic – named PHBV + HA.

### Scaffolds Characterization

**FIGURE 1:**
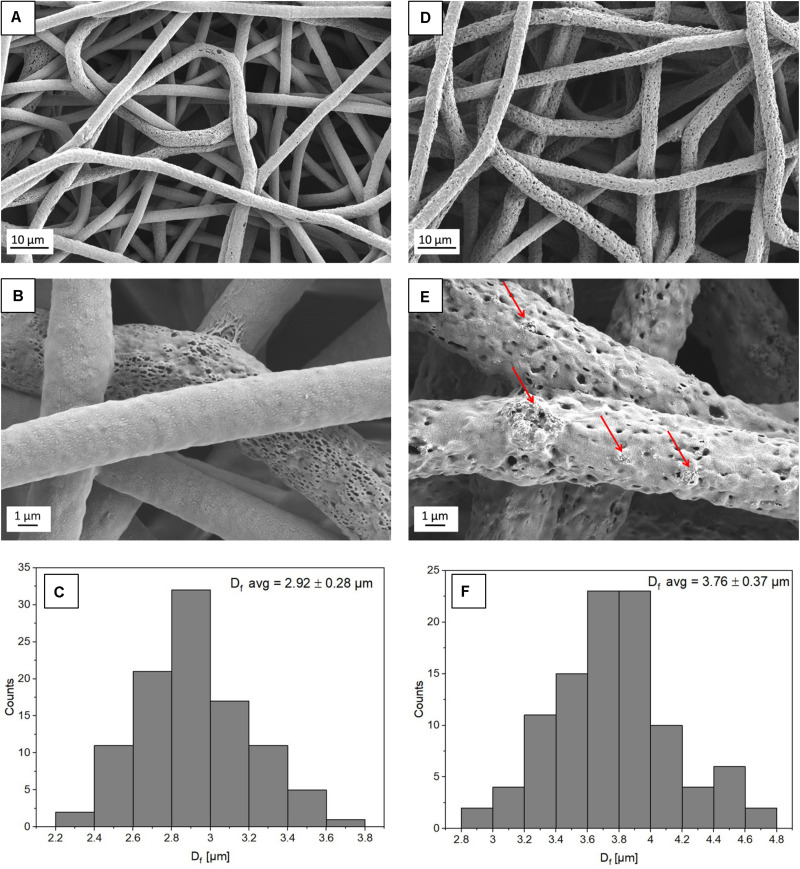
SEM micrographs showing the morphology of **(A,B)** PHBV fibers and **(D,E)** PHBV + HA hybrids. The red arrows indicate HA particles; **(C,F)** histograms indicate the fiber diameter distribution for PHBV fibers and PHBV with HA fibers, respectively.

**FIGURE 2:**
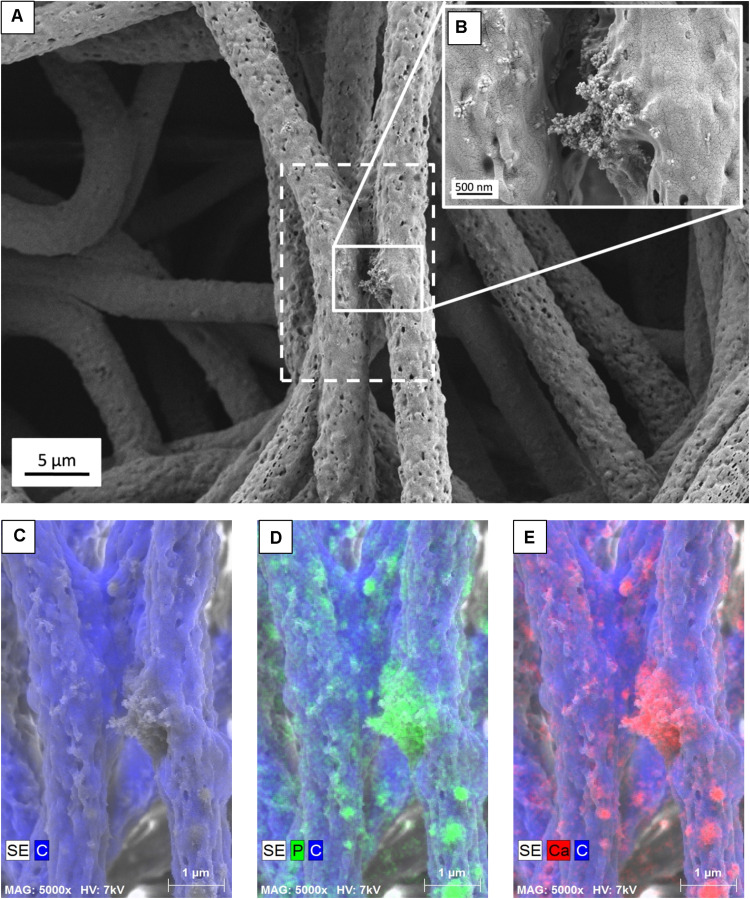
**(A)** SEM micrograph of PHBV + HA fibers with the area of EDS mapping marked with a dashed line, **(B)** Inset image showing magnified area of HA particles, **(C)** SE image with C distribution, **(D)** SE image with C and P distribution, **(E)** SE image with C and Ca distribution.

To study the morphology of the PHBV and PHBV + HA scaffolds, samples were imaged by scanning electron microscopy (SEM, Merlin Gemini II, Zeiss, Germany) at 2 kV, 100 pA, and a working distance (WD) in the range of 5–8 mm, using a secondary electrons (SE) detector. Prior to SEM observations, all samples were coated with a 5-nm Au layer using a rotary pump sputter coater (Q150RS, Quorum Technologies, United Kingdom). The average fiber diameters (D_*f*_) were measured from 100 fibers from SEM images using the ImageJ software (v. 1.51j8, United States), see histograms presented in Figure 1. Additionally, we used SEM images to estimate the spacings between fibers in scaffolds based on 10 measurements. The incorporation of HA particles into the PHBV fibers was confirmed by energy dispersive X-ray spectroscopy (EDS) using an detector (Brucker, Germany). EDS imaging and maps were done at 7 kV, 500 pA, WD = 8 mm, and a collecting time of 30 min. The merged images of the SE signal with a distribution of carbon (C), calcium (Ca), and phosphorous (P) can be seen in Figure 2. Additionally, individual fibers of the PHBV + HA sample were sliced by a focused ion beam (FIB) at 30 kV and 50 pA using a NEON CrossBeam 40EsB (Zeiss, Germany). The exposed fiber cross-section was imaged by a energy selective backscattered (EsB) detector, at 3 kV, 50 pA, and WD = 5 nm.

### Cell Culture

The *in vitro* studies were performed on both types of samples (PHBV and PHBV + HA) using human osteoblast-like cell line MG-63 (Sigma-Aldrich, United Kingdom). Samples were cut into 15-mm diameter circles, placed in 24-well plates, and sterilized for 30 min in UV light. Cells were seeded in the scaffolds at a concentration of 2 × 10^4^ cells per 1 ml in culture media containing Dulbecco’s Modified Eagle Medium supplemented with 10% fetal bovine serum (FBS), 2% antibiotics (penicillin/streptomycin), 1% amino acids, and 1% L-glutamine (all reagents Sigma-Aldrich, United Kingdom). Samples with cells were incubated in 37°C, 90% humidity, and 5% CO_2_ atmosphere for up to 7 days. The medium was replaced every 2 days.

### Cell Viability

Cell proliferation was evaluated using the CellTiter-Blue^®^ Assay (Promega, United States) after 1, 4, and 7 days of incubation, tissue culture polystyrene (TCPS) was used as control. At each time point media was replaced with 1 ml of media containing 10% of CellTiter-Blue^®^ reagent and incubated for 4 h at 37°C. From each well, 100 μm of reagent was transferred to a 96-well plate in triplicates and fluorescence was read at 560/590 nm using the microplate reader GloMax^®^ Discover System (Promega, United States).

### Cell Imaging: Confocal and SEM

After 1, 4, and 7 days of cell growth, one sample of the PHBV and PHBV + HA scaffolds were fixed with 4% paraformaldehyde (Sigma-Aldrich, United Kingdom) for 30 min. The samples were washed three times with phosphate-buffered saline (PBS, Biomed Lublin, Poland) before and after application of a fixative. Subsequently, samples were incubated in 0.1% Triton X-100 for 5 min, followed by washing in PBS and next incubated in 3% bovine serum albumin (BSA, Sigma-Aldrich, United Kingdom) for 30 min. After washing in PBS, the samples were incubated for 1 h in Alexa Fluor^TM^ 488 Phalloidin (1:400, Thermo Fisher Scientific, United States), then washed three times in PBS and finally stained with DAPI (Sigma-Aldrich, United Kingdom) for 5 min (1:1000) and again washed three times with PBS. The images were acquired using a Zeiss LSM 900 confocal microscope (Zeiss, Germany). In all the experiments, the Plan-Apochromat 63x/1.4 Oil DIC M27 objective was used. Imaging was performed using the 405 nm or 488 nm or 633 nm laser lines for exciting DAPI, Alexa Fluor^TM^ 488, and PHBV fibers, respectively. The z-stacks were recorded with the 0.4 μm step.

Cells on PHBV and composite PHBV + HA scaffolds were fixed after 1, 4, and 7 days of growth with 2.5% glutaraldehyde (Sigma Aldrich, United Kingdom) for 1 h at 4°C. Afterward, they were washed three times with PBS and dehydrated in series of ethanol (Avantor, Poland) solutions, with increasing amounts of alcohol (50, 70, 90, and 100%). Each sample was incubated in each ethanol solution for 5 min and twice in the 100% solution, followed by incubation in HMDS (Sigma-Aldrich, United Kingdom) under a fume hood until the complete evaporation of the solvent. The samples were mounted in Al holders by carbon tape and gold-sputtered with a 5 nm layer. Samples were imaged by SEM, with the settings previously described for scaffolds characterization.

### Statistics

Statistical analysis of cell proliferation was done using one-way Analysis of variance (ANOVA) followed by Tukey’s *post hoc* test in Origin Pro (ver. 2020b, United States). Differences were considered statistically significant when *p* < 0.05.

## Results

### Scaffold Characterization

From electrospinning, we obtained randomly oriented fibers with an average diameter of 2.92 ± 0.28 μm for PHBV and 3.76 ± 0.37 μm for PHBV + HA, see Figure 1. The electrospun PHBV fibers were characterized by smooth morphology, with few porous fibers in the network (Figures 1A,B). Whereas, the addition of HA particles caused a 30% increase in fiber diameter, and fibers’ morphology was more porous and rougher (Figures 1D,E). The large diameter of fibers created large spacing between individual fibers, in many cases exceeding 40 μm (see Figure 1D), allowing us to obtain a 3D scaffold with a highly porous structure.

**FIGURE 3:**
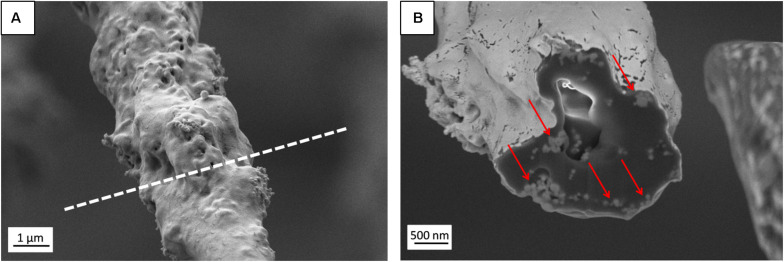
**(A)** SEM micrograph (SE detector) of individual PHBV + HA fiber with the region of FIB cutting marked by a dashed line, **(B)** Image of fiber cross-section (EsB detector) with HA particles indicated by red arrows.

The presence of HA particles was confirmed by the EDS mapping as well as imaging with the EsB detector at the fiber cross-section. HA particles were uniformly distributed within the scaffold structure, both on the surface of fibers (Figures 1E, 2) and inside fibers (Figure 3). They tended to form local aggregates as can be seen in the high magnification image in Figure 2A. Via EDS mapping (Figure 2), we confirmed that particles visible in the SEM images were composed of Ca and P (main elements of HA), whereas the main component of the fibers was carbon. Imaging with the EsB detector provided compositional contras where variation in grayscale within the sample structure allowed for the identification of different compounds. Therefore, bright spots visible inside the fiber (Figure 3B) can be identified as HA particles, since Ca and P are heavier elements than C which is found in the PHBV fibers. Particles near to the fiber border change the morphology from mostly smooth, this was observed for PHBV fibers, to highly irregular and porous fibers observed for the PHBV + HA scaffold, see Figures 1B,E. Multiple small, surface pores with a diameter below 100 nm were present along the hybrid fibers. Additionally, we noticed larger pores inside the PHBV + HA fibers with sizes up to 500 nm (Figure 3B).

### Cell Culture

**FIGURE 4:**
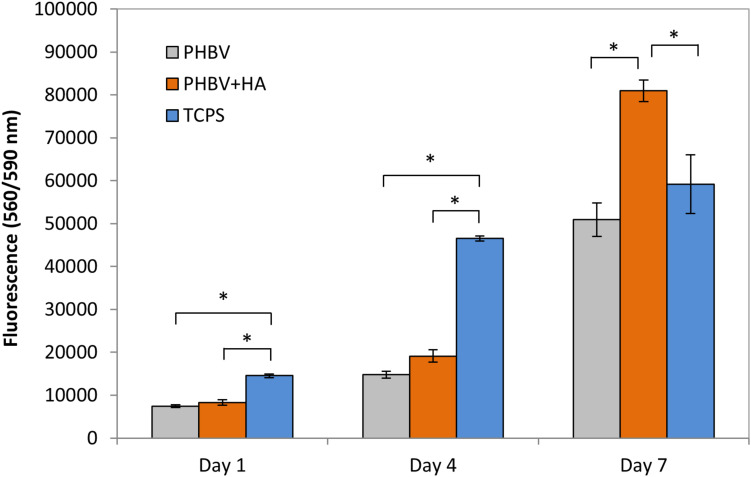
Cell proliferation based on a CellTiter-Blue^®^ assay. *statistical significance calculated with ANOVA followed by Tukey’s post hock tests, *p* < 0.05.

Using a CellTiter-Blue^®^ assay we could monitor cells proliferation over 7 days of incubation on PHBV and PHBV + HA scaffolds and control surface of TCPS, see Figure 4. This assay contains high purity dark blue resazurin, which is reduced by viable cells to pink resorufin. The fluorescent signal is proportional to the number of viable cells. After 1 day of cell incubation, the number of cells was at the same level with no statistically significant difference, for PHBV and PHBV + HA scaffolds. However, a significantly higher number was found in the TCPS control. Notably, cells required more time to attach and multiply in the 3D structure of both scaffolds while the flat surface of modified PS was specially prepared to favor cell growth. After 4 days of incubation, we observed a high proliferation of cells on TCPS and only moderate proliferation on both scaffolds. After 7 days, a higher number of cells on the PHBV + HA scaffold compared to the PHBV scaffold and control was detected. In the control sample after this time, cells occupied all available surfaces, so their multiplication was slowed down; while the 3D structure of the PHBV + HA scaffolds provided much more space for cells to grow and spread.

**FIGURE 5:**
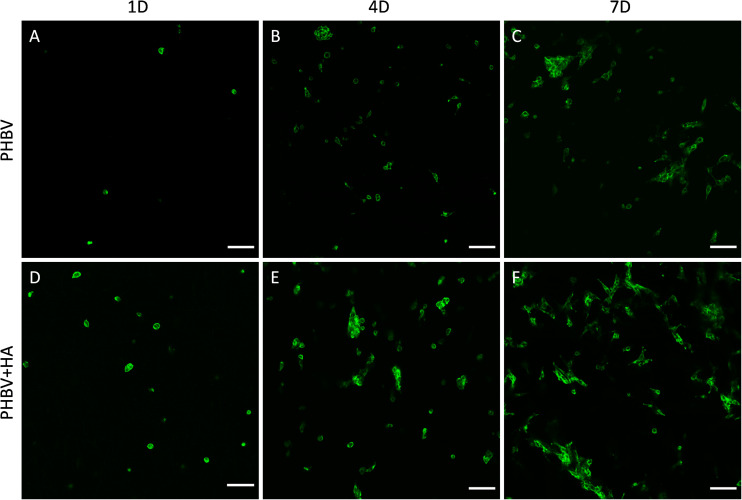
Confocal images of cell spreading on **(A–C)** the PHBV and **(D–F)** PHBV + HA scaffolds after 1, 4, and 7 days (1D, 4D, and 7D) of incubation, actin fibers stained with Alexa Fluor^TM^ 488 Phalloidin (green), scale bar 100 μm.

Our confocal microscopy observations confirmed the results of the proliferation test, showing constant multiplication of cells (an increase of their number) on both tested materials over the incubation period, see Figure 5. Additionally, a higher cell number was observed on the PHBV + HA scaffolds at each time point (Figures 5D–F). In Figure 5, we show only the actin fiber staining corresponding to cell spreading and relative quantity over a large imaging area. In the Supporting Information file, we provide a similar figure including the autofluorescence from PHBV fibers, see Supplementary Figure 1.

**FIGURE 6:**
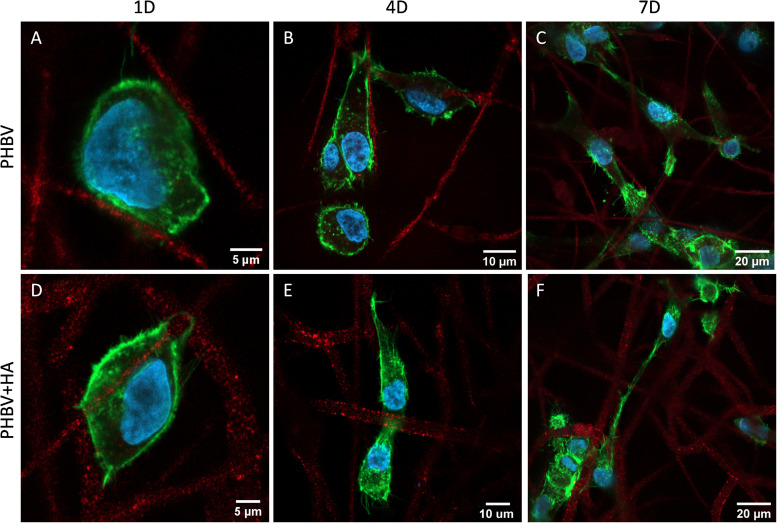
Confocal images showing cell morphology on **(A–C)** the PHBV and **(D–F)** PHBV + HA scaffolds after 1, 4, and 7 days (1D, 4D, and 7D) of incubation; nucleus stained with DAPI (blue); actin fibers stained with Alexa Fluor^TM^ 488 Phalloidin (green); autofluorescence of PHBV fibers (red).

Figure 6 presents the morphology of cells growing on PHBV and PHBV + HA scaffolds. Using the laser with a wavelength of 633 nm, we observed the autofluorescence of PHBV fibers. Therefore, while performing confocal imaging we could also include fibers in the images, allowing us to observe the arrangements of the cells in the context of the 3D scaffold structure. After 1 day of incubation, the cells were mainly round in shape, but starting to form some filopodia to reach adjacent fibers, especially in the PHBV + HA scaffold. After 4 days, the cell shape become elongated, osteoblasts were reaching distant fibers, and in many cases, overlapping them. The increased number of filopodia allowed cells to explore the 3D scaffolds’ structure and create cell-to-cell interactions. After 7 days, many more cells were visible within the imaging area (see Figure 6F). They tended to be stretched along fibers; however, they were also forming multiple connections and filopodia in all directions. In the case of the PHBV + HA scaffold, we could observe cells forming long structures above 40 μm in length to communicate with each other.

**FIGURE 7:**
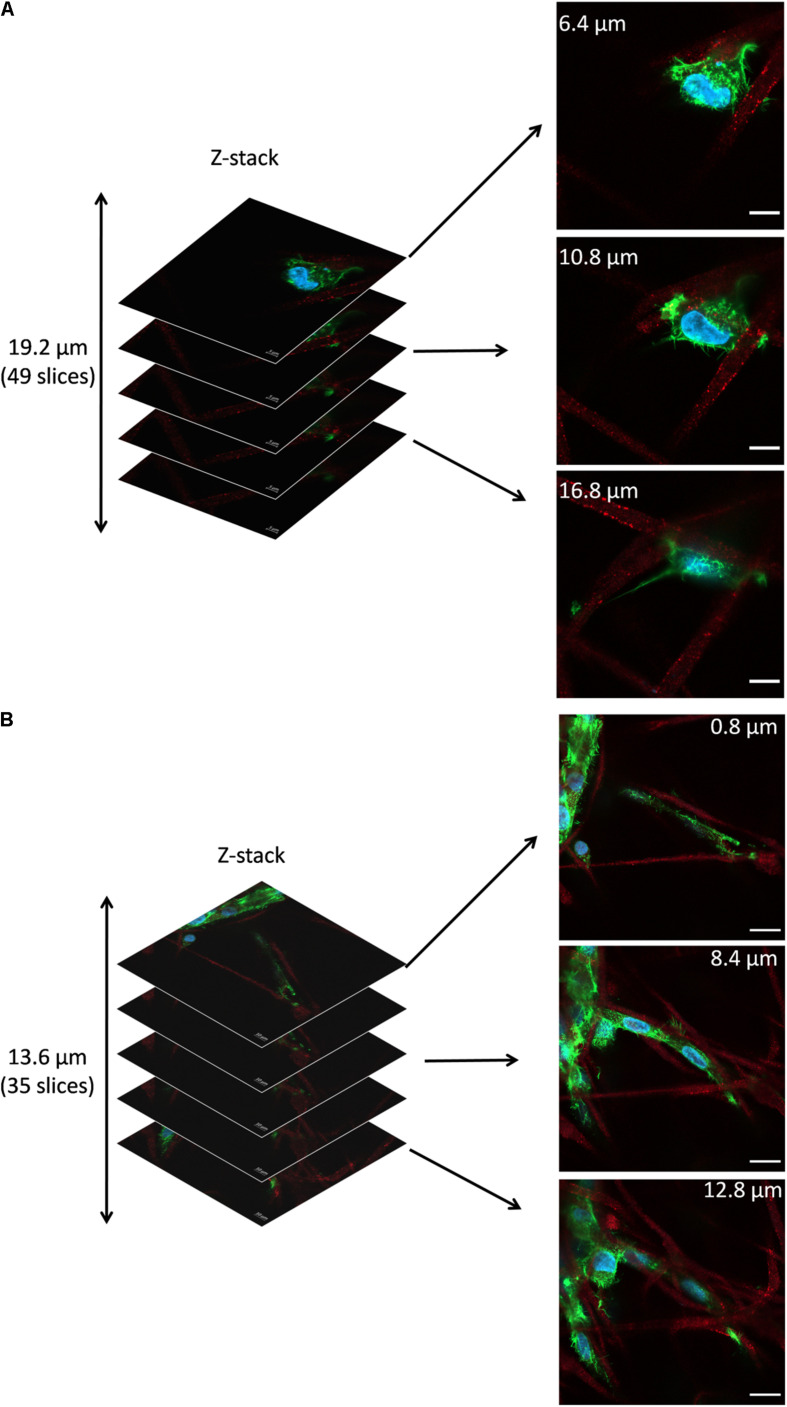
Z-stacks of confocal images: **(A)** Cell in PHBV + HA scaffold after 4 days of incubation, scale bar 10 μm, **(B)** Cells in PHBV + HA scaffold after 7 days of incubation, scale bar 20 μm.

Figure 7 shows the representative z-stacks showing cell integration with PHBV + HA scaffolds after 4 and 7 days of incubation. Z-stacks in confocal microscopy enable the collection of a series of consecutive images to study the whole cell structure with all protrusions, also reaching deep into the scaffold structure. In the case of cells growing on flat surfaces, like glass slides or TCPS, they create focal adhesion points and explore the environment in one horizontal plane. Whereas, cells growing on 3D scaffolds make connections in all possible directions, especially if the porosity allows them to migrate inside the material. In the case of the example osteoblasts growing on the PHBV + HA scaffold for 4 days (Figure 7A), the main cell body was located between three neighboring, intersecting fibers and the cell formed multiple filopodia to attach and sense them. Imaging deeper into the scaffold structure, we could observe that the cell was additionally forming an approximate 30-μm long attachment to another fiber. Therefore, the osteoblast was reaching 19.2 μm into the depth of the PHBV + HA scaffold. After 7 days of incubation, cell proliferation on the PHBV + HA scaffold was very high, therefore, multiple daughter cells close to each other were detected, see Figure 7B. Osteoblasts were mostly growing along the hybrid fibers, however, they created multiple adhesions to other nearby fibers. The whole structure shows that cells were intertwined between the fibers attaching to them from all directions. As we moved deeper into the PHBV + HA scaffold more osteoblasts were visible in the center and left bottom of the imaged area, see Figure 7B. This z-stack covers the thickness of 13.6 μm, but more cells were present below and above the imaged volume.

**FIGURE 8:**
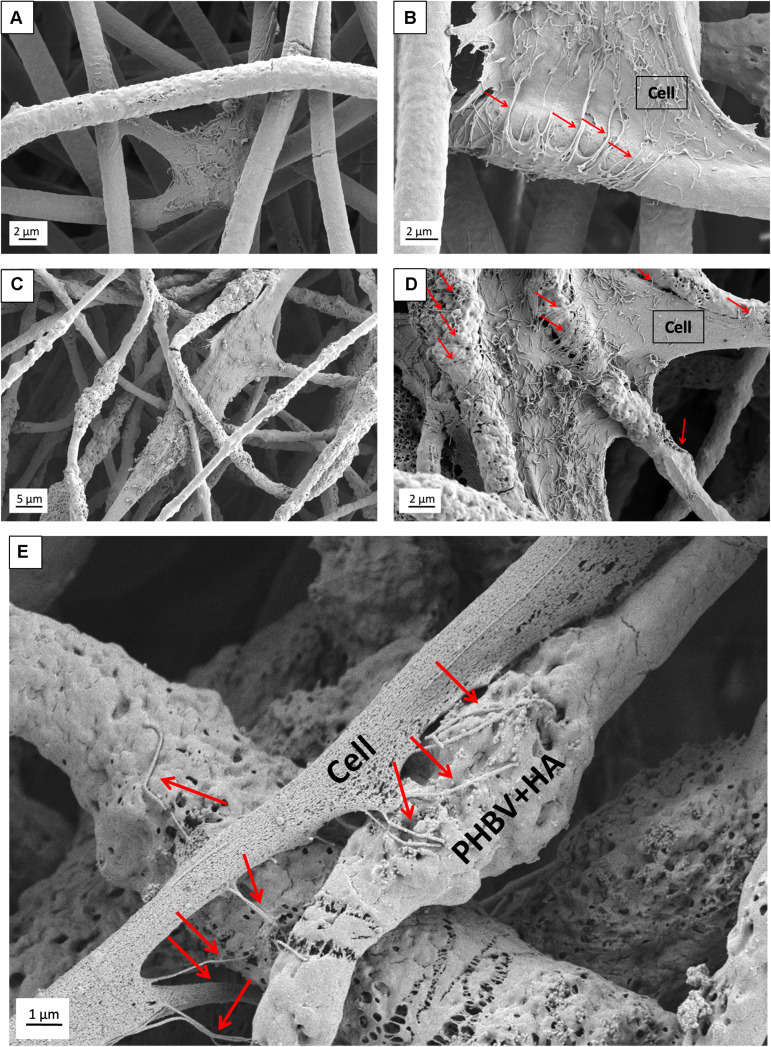
SEM micrograph SE detector showing cell morphology after 7 days of incubation on: **(A,B)** PHBV fibers, **(C,D)** PHBV + HA fibers; **(E)** higher magnification micrograph showing in detail the filopodia attachment to the rough and porous surface of PHBV fibers with HA particles exposed on their surfaces. Red arrows indicate filopodia interacting with fibers.

**FIGURE 9:**
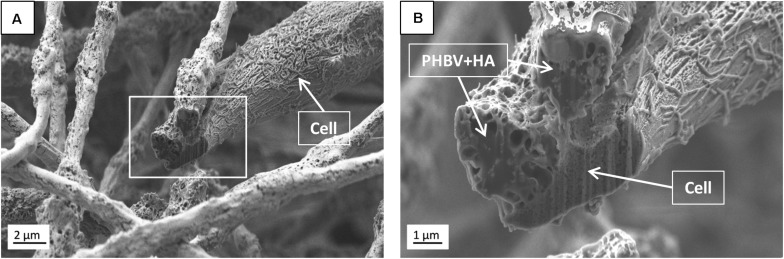
SEM micrographs (SE detector) of **(A)** the cross-section performed using FIB to visualize the cell ingrowth between PHBV + HA fibers after 7 days of incubation, **(B)** higher magnification, enlarging the square from **(A)**.

Additionally, SEM imaging was performed to reveal in great detail the cell integration with electrospun scaffolds, and to verify cell adaptation to variation in the surface morphology of fibers. The interactions at the interfaces between fibers and osteoblasts growing for 7 days in the PHBV and PHBV + HA scaffolds are shown in Figure 8. Cells that grew on smooth PHBV fibers were spreading between and adhering to many surrounding fibers (Figure 8A). Often cells were overlapping PHBV fibers with filopodia, see Figure 8B. From the microscopy observation, we noticed that cells on the PHBV + HA fibers were elongated reaching more distant fibers than those on PHBV fibers, as well as perfectly occupying small spaces in between fibers, see Figure 8C. Importantly, the rough and porous surface of PHBV + HA fibers with exposed ceramic particles provided multiple anchoring points for cells and encouraged the formation of sensing filopodia which are visible in Figure 8E. Moreover, we observed much more deposits and fibrils on the surface of cells growing on the PHBV + HA scaffold (Figures 8C,D) compared to solely polymer fibers (Figures 8A,B). These collagen fibrils are typical for an early stage of ECM formation (Metwally et al., 2019). In this study, both types of PHBV electrospun scaffolds were highly porous with large spacing between fibers. Therefore, while seeding cells only a few of them attached to the topmost fibers, whereas the majority fell deeper and attached, spread, and proliferated throughout. This has been proven by deep confocal imaging inside the scaffolds’ structures as presented in Figure 7. Additionally, with FIB-SEM, by slicing the samples we could visualize the cross-section of cell integration in between the PHBV + HA fibers, see Figure 9. The imaged cell was perfectly stretched along the fibers as shown in Figure 9A, but it formed an additional anchoring point using the small gap between fibers’ crossing as indicated by the higher magnification SEM micrograph in Figure 9B.

## Discussion

### Scaffold Characterization

We successfully produced two types of scaffolds with two distinct morphologies affecting the cell spreading, adhesion, and proliferation. The obtained diameter of PHBV fibers was close to the value of 2.79 ± 0.2 μm, as previously reported (Kaniuk et al., 2020). The morphology and size of fibers prepared via electrospinning were affected by the polymer solution, environmental conditions, and applied electric field (Krysiak et al., 2020; Szewczyk et al., 2020). It was followed by mechanical (Ura et al., 2020) and surface properties (Metwally et al., 2020). Therefore, PHBV-based scaffolds described in Suslu et al. (2014) had significantly smaller diameters of 571 ± 160 nm compared to our study, however, similarly, they obtained increased fiber diameter with the addition of HA particles. The addition of an inorganic filler can cause either an increase or decrease in electrospun fiber diameter depending on particle size as well as the viscosity and conductivity of suspension. Augustine et al. (2020) observed a slight decrease of fiber diameter compared to solely polymer fibers for fibers containing PHBV and CeO_2_ nanoparticles (diameter below 10 nm). However, the described results were flawed by a large error as the standard deviations were near or higher than average values. Whereas, Kouhi et al. (2015, 2018) reported a slight increase in fiber diameter for electrospun composites of PHBV-bredigite as well as PHBV + HA compared to PHBV scaffolds (bredigite particle size was 25 nm and HA 23 nm). Different findings were described by Paşcu et al. (2013) for PHBV fibers with HA nanoparticles. Fibers electrospun from 15% PHBV solution had diameters over 9 μm, whereas after adding 5% of HA to the polymer solution the resulting fiber diameter was three times higher and reached over 18 μm. Porous fibers are most often obtained by the careful selection of solvents as well as high humidity during electrospinning due to phase separation (Metwally et al., 2020; Szewczyk and Stachewicz, 2020). A different approach was described by Lyu et al. (2016) for blended PHB with polyethylene oxide (PEO) fibers followed by immersion in SBF for 4 weeks causing PEO leaching and pore formation on fibers. In the case of our electrospun PHBV fibers, the porosity was caused by the vapor-induced phase separation (VIPS) (Huang and Thomas, 2020; Szewczyk and Stachewicz, 2020), which was enhanced by the addition of hygroscopic HA particles, which was also observed and described for electrospun PHBV with the addition of PLLA (Wagner et al., 2014).

Often when blend electrospinning from a suspension containing organic and inorganic compounds, the applied particles are entirely embedded inside the polymer fibers (Bai et al., 2015; Sadat-Shojai, 2016; Kouhi et al., 2018). Whereas, using the post-processing of electrospun scaffolds for fiber decoration with ceramic particles by spraying or precipitation after soaking in simulated body fluid (SBF) solution results in particles only covering fibers (Gupta et al., 2009; Ramier et al., 2014). The imaging of particle distribution within electrospun fibers is challenging. SEM provides excellent information about the morphology of electrospun materials, therefore particles occurring on the surface of the fiber can be easily imaged (Ramier et al., 2014; Suslu et al., 2014), while embedded particles can be identified by thickened fiber areas, similar to beads (Sadat-Shojai, 2016; Kouhi et al., 2018). Transmission electron microscopy (TEM) is often used to investigate a few hundred-nanometer fibers, resulting in 2D images, which in some cases can give inconclusive information on whether particles are outside or inside fibers (Ramier et al., 2014). Various microscopy techniques complement each other thus applying several of them is necessary to obtain complete structural information about hybrid materials. Additional insights into material structure and particle distribution can be acquired from FIB-SEM cross-sections, as shown in Figure 3. Furthermore, based on the collection of consecutive SEM images registered after FIB cutting and subsequent reconstruction of the investigated area, it is possible to obtain 3D information regarding particle shape and position inside electrospun fibers (Cherpinski et al., 2019). Particles included inside the fibers affect their mechanical properties by increasing elastic modulus and tensile strength, changing their stiffness, and creating a more rigid scaffold (Ramier et al., 2014; Kouhi et al., 2018; Ivanoska-Dacikj et al., 2020), but the availability of the bioactive compound is limited since they are inside and dependent on polymer degradation. A too high concentration of ceramic filler in blend electrospinning (Kouhi et al., 2018; Ivanoska-Dacikj et al., 2020) can deteriorate mechanical properties, even below values for solely polymer fibers. A similar effect of decreased mechanical performance was observed for fibers covered with an excess of particles, like in the case of applying electrospraying (Ramier et al., 2014). Therefore, careful optimization of the amount as well as the distribution of particles within hybrid fibers is needed to prepare desired scaffold-supporting bone tissue regeneration. An advantage of our composite organic-inorganic system is the presence of HA particles both outside and inside the electrospun fibers obtained within a single-step method.

### Cell Culture

Scaffolds’ architecture, surface properties as well as composition are crucial to guide bone cell attachment, proliferation, and maturation toward tissue regeneration (Zhu et al., 2020). It was proven that the moderate surface roughness of electrospun fibers created by beads on the fibers (Luo et al., 2012) or fibers porosity (Metwally et al., 2020) can promote initial bone cell adhesion and spreading followed by proliferation and differentiation. Additional improvement can be achieved by adding ceramic particles into the polymer network, therefore, reinforcing the scaffold with bioactive compounds which better match the mechanical and biological properties of bone tissue (Li and Chang, 2004; Bai et al., 2015; Wu et al., 2017). In our work, increased cell proliferation on the hybrid PHBV + HA scaffold is attributed to irregular fibers with surface porosity and the availability of HA particles. All together they contribute to structural signaling for cells with nano- and micro-roughness as well as biological signaling with the release of Ca and P ions. Cells in contact with the complex 3D structure of electrospun scaffolds need more time to form strong adhesion points and spread, only after they are settled, can the boost in proliferation occur. After the initial moderate cell proliferation, we observed a significant increase between the 4th and 7th day of cell culture on both PHBV-based scaffolds. Similar observations were previously reported for mouse pre-osteoblasts (MC3TC-E1) growing on PHB/HA hybrids (Sadat-Shojai, 2016). Even more time was needed for SaOS-2 cells growing on HA/PHBV scaffolds, where increased cell proliferation was noted between the 7th and 10th days of incubation (Suslu et al., 2014). For successful bone tissue regeneration, scaffolds with the right mechanical performance must also provide sufficient porosity for inside cell infiltration as well as nutrient and metabolite flow (Zhu et al., 2020). Electrospun membranes with a fiber diameter below 1 μm result in a scaffold with small pore sizes, even though the porosity of the whole structure is very high. In such a case, the scaffold serves as a 2D structure limiting cell ingrowth so they only form a surface layer (Luo et al., 2012; Ura et al., 2019). In this study, fiber diameters in both the PHBV-based scaffolds exceeded 2.5 μm with large pores, resulting in cell attachment and proliferation deep inside the structure, as we show on the z-stack confocal images in Figure 7. Also, the large spacing between fibers promoted cell elongation, stretching, and long cellular protrusion formation to bridge the gap between neighboring fibers and establish cell-to-cell communication. For cells, topography sensing is how they interact with the environment, which is done through filopodia formation (Albuschies and Vogel, 2013). The structure of electrospun PHBV-based scaffolds with micrometer-sized fibers provided cells with micro-features that they overlapped while establishing cell-material connections, as can be seen in Figures 8B,D. Whereas, the presence of HA particles and irregular fibers in the PHBV + HA scaffold additionally created nano-roughness that enhanced sensing filopodia formation to explore the material structure by cells, thus cell anchoring occurred as illustrated in Figure 8E.

Scaffolds in tissue engineering are designed to play a temporary supportive role, enabling cell attachment and guiding tissue development. After fulfilling its task, scaffolds should degrade to the biocompatible products and at the same time cells should secrete their own ECM. The collagen fibrils formation on the surface of osteoblasts followed by early mineralization is a typical sign of cell development toward bone tissue regeneration. These events can be enhanced by various signals: biological growth factors (Borsani et al., 2018), the addition of bioactive mineral particles (Kouhi et al., 2018), or scaffolds’ properties like surface charge (Szewczyk et al., 2019a). In our study fibers’ roughness, porosity, and HA presence in electrospun hybrid PHBV + HA scaffolds enhanced early collagen and mineral deposit formation on osteoblasts after 7 days of incubation, compared to PHBV scaffolds, as we can see in Figure 8.

### Conclusion

This study represents a simple way of obtaining hybrid scaffolds by blend electrospinning a PHBV solution with HA particles without any post-processing. We successfully electrospun PHBV + HA and obtained a rough morphology of the fibers that eventually increased cell anchorage and proliferation. HA nanoparticles were well-adhered to the surface but were also present inside fibers, which is crucial for longer cell culture studies. We have proven this with EDS and FIB-SEM analysis. The combination of organic-inorganic components in a scaffold affects not only cell development and mobility but also ECM formation in the form of collagen fibrils and mineral deposits on their surface. This is one of the most desired processes that researchers want to obtain in bone tissue regeneration within such short-term studies. Importantly, the high porosity and distance between fibers (pore sizes in our scaffolds) allowed for the cell deep integration with fibers which has been shown via z-stack confocal imaging. We successfully created a suitable 3D environment for bone regeneration processes which is proven by cell integration and enhanced anchoring within the whole scaffold structure. These results show the great potential in the development of hybrid scaffolds which stimulate and intensify filopodia formation thus promoting desired specific cell activities in tissue regeneration processes.

## Data Availability Statement

The original contributions presented in the study are included in the article/Supplementary Material, further inquiries can be directed to the corresponding authors.

## Author Contributions

JEK and US contributed to the conception and design of the study. ŁK and JEK performed the electrospinning. JEK performed the cell culture, SEM imaging, statistical analysis, and wrote the first draft of the manuscript. KB completed the confocal microscopy imaging. AG performed the FIB-SEM microscopy. KB and US wrote the sections of the manuscript. All authors contributed to the manuscript revision, read, and approved the submitted version.

## Conflict of Interest

The authors declare that the research was conducted in the absence of any commercial or financial relationships that could be construed as a potential conflict of interest.
