# Supplementary material for: Enhanced Cells Anchoring to Electrospun Hybrid Scaffolds With PHBV and HA Particles for Bone Tissue Regeneration
Source: Front Bioeng Biotechnol. 2021 Feb 17;9:632029. doi: 10.3389/fbioe.2021.632029 (PMC7928304; doi:10.3389/fbioe.2021.632029)
Supplement: Supplementary file 1 [file Data_Sheet_1.pdf]

Supporting Information to

# Enhanced cells anchoring to electrospun hybrid scaffolds with PHBV and HA particles for bone tissue regeneration

**Joanna E. Karbowniczek*, Łukasz Kaniuk, Krzysztof Berniak, Adam Gruszczyński, Urszula Stachewicz***

*Faculty of Metals Engineering and Industrial Computer Science, AGH University of Science and Technology, Cracow, Poland*

**Corresponding authors email:** jkarbow@agh.edu.pl; ustachew@agh.edu.pl

In this supporting information we provide the confocal images showing the cell and electrospun PHBV fibers (Figure S1) and SEM images showing cell extension on PHBV+HA scaffold (Figure S2).

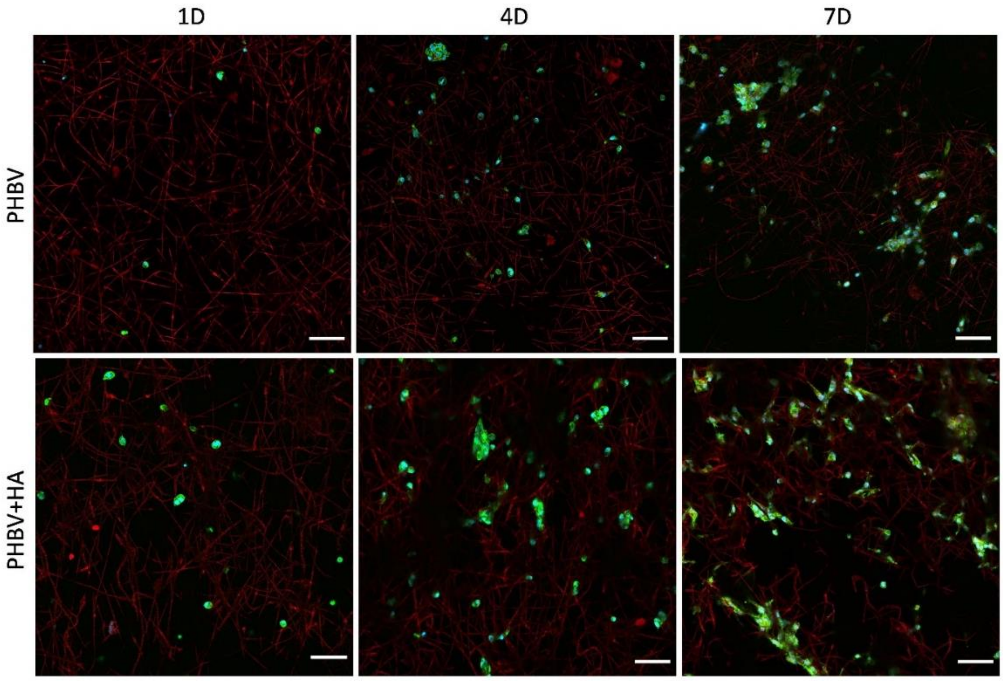


**Figure S1**. Confocal images of cell spreading on (**A-D**) the PHBV and (**D-F**) PHBV+HA scaffolds after 1, 4 and 7 days (1D, 4D, 7D) of incubation, green – actin fibers stained with Alexa Fluor™ 488, scale bar 100 µm. The autofluorescence of PHBV fibers is shown in red.

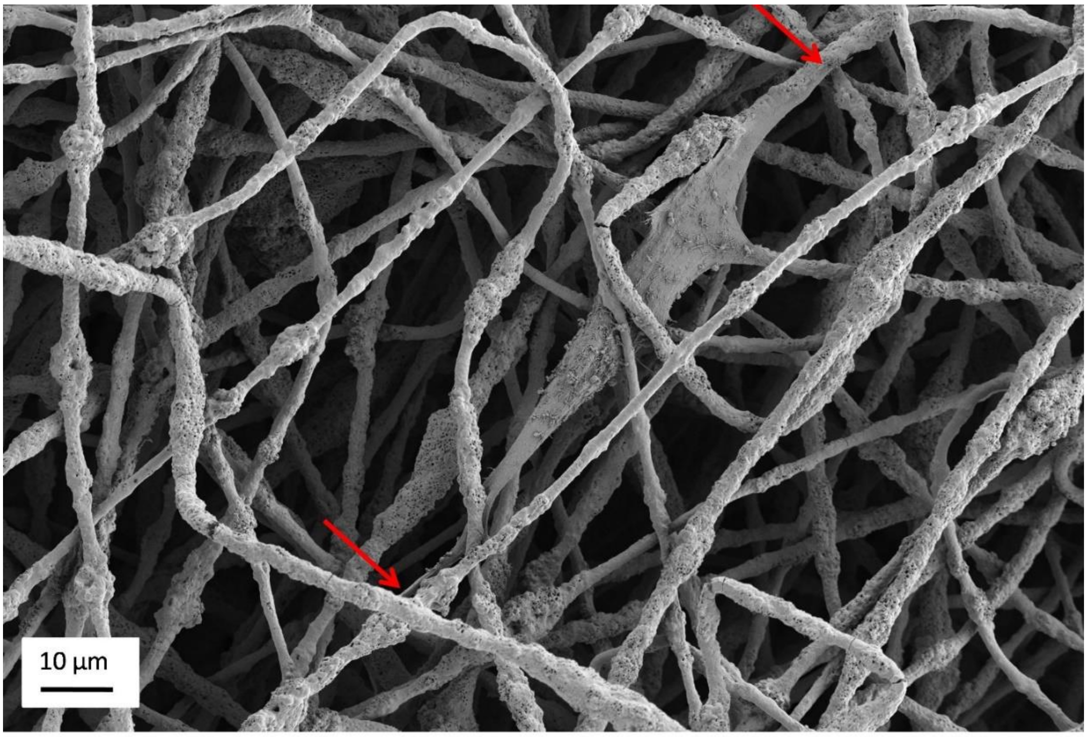


**Figure S2.** SEM micrograph showing cell extension over 70 µm without support to reach another distant anchoring point in the PHBV+HA scaffold. The arrows indicate the ends of the cell. Near to upper arrow, the cell is growing along the fiber. Whereas, in the middle of the cell we could observe two fiber above cell and additional fiber just below it, showing the cell ability to growing in the small spacing between fiber in PHBV scaffolds .
